# The Restorative Effects of *Eucommia ulmoides* Oliver Leaf Extract on Vascular Function in Spontaneously Hypertensive Rats

**DOI:** 10.3390/molecules201219826

**Published:** 2015-12-09

**Authors:** Shingo Hosoo, Masahiro Koyama, Mai Kato, Tetsuya Hirata, Yasuyo Yamaguchi, Hiroo Yamasaki, Atsunori Wada, Keiji Wada, Sansei Nishibe, Kozo Nakamura

**Affiliations:** 1 R & D Center, Kobayashi Pharmaceutical Co., Ltd., 1-30-3, Toyokawa, Ibaraki, Osaka 657-0057, Japan; s.hosoo@kobayashi.co.jp (S.H.); y.yamaguchi@kobayashi.co.jp (Y.Y.); h.yamasaki@kobayashi.co.jp (H.Y.); a.wada@kobayashi.co.jp (A.W.); 2 Department of Bioscience and Biotechnology, Graduate School of Agriculture, Shinshu University, 8304, Minamiminowa, Nagano 399-4598, Japan; mkoyama32@shinshu-u.ac.jp (M.K.); 15aa310g@shinshu-u.ac.jp (M.K.); 3 Department of Pharmaceutical Sciences, Health Sciences University of Hokkaido, 1757 Kanazawa, Tobetsu-cho, Ishikari, Hokkaido 061-0293, Japan; wadakg@hoku-iryo-u.ac.jp (K.W.); nishibe@hoku-iryo-u.ac.jp (S.N.)

**Keywords:** *Eucommia* leaf extract, ACh-induced endothelium-dependent relaxation, nitric oxide, aortic media thickness, spontaneously hypertensive rat

## Abstract

*Eucommia ulmoides* Oliv. leaf is a traditional Chinese antihypertensive and antidiabetic medicine. We examined the effects of chronic *Eucommia* leaf extract (ELE) administration on artery function and morphology in spontaneously hypertensive rats (SHRs). ELE was orally administered via normal diet *ad libitum* to six-week-old male SHRs at a concentration of 5% for seven weeks. Acetylcholine (ACh)-induced endothelium-dependent relaxation, sodium nitroprusside (SNP)-induced endothelium-independent relaxation, plasma nitric oxide (NO) levels, and media thickness were assessed. ELE significantly improved ACh-induced aortic endothelium-dependent relaxation but did not affect SNP-induced endothelium-independent relaxation in the SHRs, as compared to the animals receiving normal diet. Plasma NO levels and media thickness were significantly increased and decreased, respectively, in the ELE-treated SHRs. Therefore, long-term ELE administration may effectively improve vascular function by increasing plasma NO levels and bioavailability, and by preventing vascular hypertrophy in the SHR aorta.

## 1. Introduction

According to a World Health Organisation report, cardiovascular disease (CVD), including heart attacks, heart failure, kidney disease, and stroke, accounts for approximately 17 million deaths per year, corresponding to almost one-third of total global mortality [1]. Hypertension is one of the key risk factors for CVD and contributes to at least 45% of CVD-attributed deaths and to 51% of those attributed to stroke [1]. Vascular dysfunction is directly linked to an increased risk for CVD [2,3]. Therefore, maintenance of vascular function is important in the prevention of CVD as well as in hypertension management.

The vascular endothelium plays an important role in the regulation of vascular tone, tissue blood flow, inflammatory responses, and maintenance of blood fluidity [4,5]. Vasodilatory and antiproliferative endothelium-derived factors include nitric oxide (NO), endothelium-derived hyperpolarizing factor [6], and prostacyclin I_2_ [7]. NO is a particularly crucial factor in vascular homeostasis. Indeed, endothelial dysfunction is characterized by reduced NO production or activity and increased concentrations of endothelium-derived contracting factors [8]. Endothelial function may reflect an individual’s lifestyle, as well as their propensity to develop atherosclerotic disease; the presence of endothelial dysfunction is considered to represent an important initial step in the development of atherosclerosis [9].

*Eucommia ulmoides* Oliver is the only known species of the genus *Eucommia*, which is the only genus in the Eucommiaceae family. The bark of *E. ulmoides* (*Cortex Eucommiae*) is used to produce herbal medicines that are traditionally used as analeptic, analgesic, sedative, antihypertensive, and diuretic drugs [10]; it is also used as a crude drug in Japan. Since the 1970s, *Eucommia* leaves (ELs) have been used in the Sichuan District of China as an antihypertensive drug and a health food [11]. In Japan, ELs are used in traditional beverages for health promotion. An EL product called “Tochu-cha” in Japanese is commercially available as a government-approved Food for Specified Health Uses for people with higher than normal blood pressure.

Previously, several pharmacological studies reported that EL extract (ELE) also exhibited antihypertensive [12], antihyperlipidemic [13], antioxidant [14], antihyperglycemic [15], insulin-sensitizing [16], and anti-obesity effects [17,18]. In particular, the antihypertensive effects of ELE were reported by *in vivo* and *in vitro* studies, indicating that ELE exhibited a dose-dependent blood-pressure-lowering effect in the spontaneously hypertensive rat (SHR) [19] and elicited an endothelium-dependent, NO-mediated vasorelaxation of the isolated rat aorta *in vitro* [20]; long-term intake also decreased blood pressure in human subjects with high normal blood pressure and mild hypertension [21,22]. However, there is little information about the effects of long-term ELE intake on vascular function. Vessel endothelial dysfunction is a critical trigger of CVD and an independent predictor of cardiovascular events [23,24]. Chronic endothelial dysfunction results in morphological changes of the arterial wall, such as hypertrophy, and initiation of atherosclerosis. Therefore, we examined the effects of chronic administration of ELE on SHR aortic endothelial function by examining acetylcholine (ACh)-induced relaxation and plasma NO levels, and on morphological changes by immunohistochemical analysis.

## 2. Results

### 2.1. Body Weight, Food and Water Intake, and Systolic Blood Pressure (SBP)

The body weights of the rats in all groups increased over time. However, during the seven-week experimental period, no significant differences between the body weight-gain or daily food and water intake of the groups were observed (Table 1). SBP was measured by the tail cuff method and the data were expressed as ΔSBP, which was the difference between the SBP on day-1 and the SBP measured three or seven weeks after the beginning of the experiment. The ΔSBP values in the SHR-control group at weeks 3 and 7 were significantly higher (*p* < 0.01 or 0.05) than those in the Wistar Kyoto rat (WKY) group. The ΔSBP in the SHR-ELE group was also significantly higher (*p* < 0.01) than that of the WKY group. However, the ΔSBP values in the SHR-ELE group at weeks three and seven were significantly lower (*p* < 0.01 or 0.05) than those in the SHR-control group (Table 1).

### 2.2. Relaxation Response

**Figure 1:**
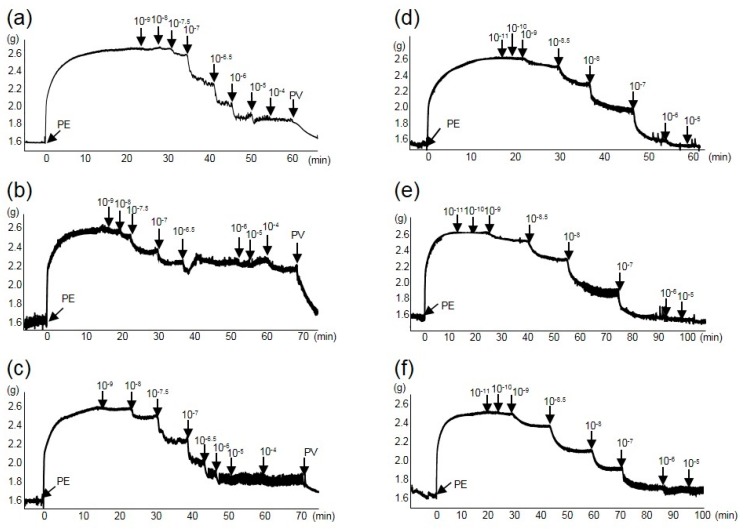
Representative acetylcholine (ACh) and sodium nitroprusside (SNP) relaxation profiles of thoracic aorta rings from the test animals. (**a**) ACh-Wistar Kyoto rat (WKY) group; (**b**) ACh-spontaneously hypertensive rat (SHR)-control group; (**c**) ACh-SHR-*Eucommia* leaf extract (ELE) group; (**d**) SNP-WKY group; (**e**) SNP-SHR-control group; and (**f**) SNP-SHR-ELE group. ACh and SNP were added cumulatively at concentrations of 10^−9^–10^−4^ M and 10^−11^–10^−5^ M, respectively, to 0.3 μM phenylephrine (PE)-contracted thoracic aorta rings. PV, papaverine.

**Figure 2:**
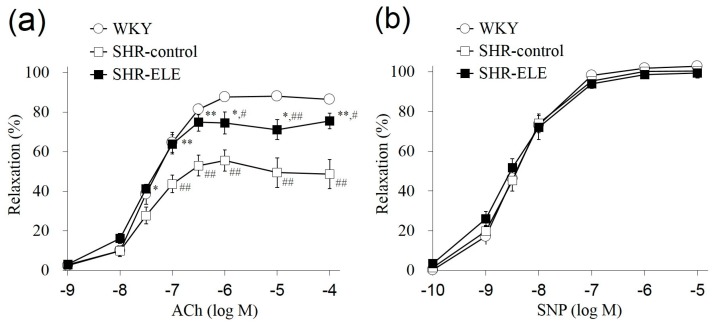
(**a**) Endothelium-dependent relaxation curve induced by acetylcholine (ACh) and (**b**) endothelium-independent relaxation curve induced by sodium nitroprusside (SNP) in thoracic aortic rings from the indicated treatment groups. The values represent the means ± SE; * *p* < 0.05, ** *p* < 0.01, as compared with the spontaneously hypertensive rat (SHR)-control group; ^#^ *p* < 0.05, ^##^ *p* < 0.01, as compared with the Wistar Kyoto rat (WKY) group. ELE, *Eucommia* leaf extract.

To investigate endothelium-dependent relaxation, we applied ACh (10^−9^–10^−4^ M) cumulatively to aortic tissues that had been pre-contracted with phenylephrine (PE, 0.3 μM). Figure 1a–c show representative profiles of ACh-induced relaxation in each group. Figure 2a shows ACh-induced relaxation curves plotted as the mean values of seven individual tests in each group. The relaxation response at 10^−7^–10^−4^ M ACh in the SHR-control group was significantly lower (*p* < 0.01) than that in the WKY group. The effective concentration causing 50% relaxation (EC_50_) in the SHR-control group was 0.61 ± 0.19 μM, which was significantly higher (*p* < 0.01) than that of the WKY group (0.076 ± 0.014 μM). In contrast, the ACh-induced relaxation response at 10^−7.5^–10^−4^ M in the SHR-ELE group was significantly higher (*p* < 0.01 or 0.05) than that in the SHR-control group. The EC_50_ of the SHR-ELE group was 0.15 ± 0.06 μM, which was significantly lower (*p* < 0.01) than that of the SHR-control group. Figure 1d–f show representative profiles of sodium nitroprusside (SNP)-induced relaxation in each group. The SNP-induced endothelium-independent relaxation and the respective EC_50_ values did not differ significantly between the three groups (Figure 2b). However, ELE intake improved endothelium-dependent vasorelaxation in the SHR thoracic aorta.

**Table 1:** Body weight, food and water intake, and the change in systolic blood pressure (SBP) during the seven-week study.

	Body Weight (g)			Food Intake (g/Day)	Water Intake (mL/Day)	ΔSBP (mmHg)	
	Day-1	Week 7	Gain			Week 3	Week 7
WKY	125.7 ± 2.2	347.5 ± 1.9	221.9 ± 3.5	18.4 ± 0.5	29.9 ± 0.8	3.6 ± 1.8	16.4 ± 4.7
SHR-control	136.1 ± 1.2	331.6 ± 7.7	195.5 ± 6.8	19.8 ± 0.4	33.2 ± 1.1	35.6 ± 0.7 ^#^	72.9 ± 2.4 ^##^
SHR-ELE	136.7 ± 0.4	321.5 ± 3.8	184.8 ± 3.7	19.2 ± 0.8	35.6 ± 1.4	17.8 ± 2.5 **^,##^	67.1 ± 2.3 *^,##^

WKY, Wistar Kyoto rat; SHR, spontaneously hypertensive rat; ELE, *Eucommia* leaf extract; ΔSBP, change in systolic blood pressure. The ΔSBP is the SBP at the indicated time-point minus the SBP on day -1. All values represent the means ± SE. * *p* < 0.05, ** *p* < 0.01, as compared with the SHR-control group; ^#^ *p* < 0.05, ^##^ *p* < 0.01, as compared with the WKY group.

### 2.3. The Effect of ELE on Plasma NO

**Figure 3:**
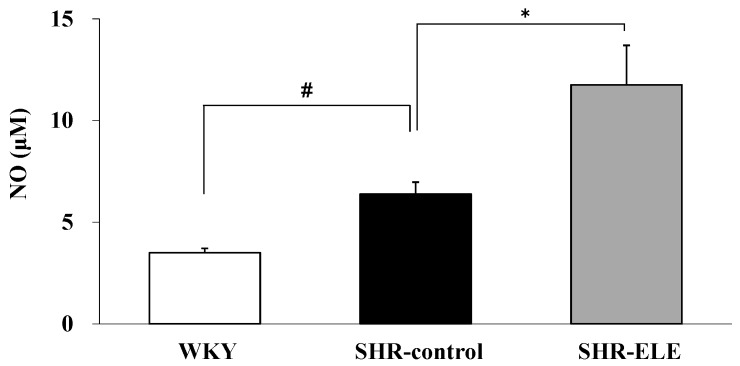
Plasma nitric oxide (NO) levels in the indicated treatment groups. The values represent the means ± SE; * *p* < 0.05, compared with the spontaneously hypertensive rat (SHR)-control group; ^#^ *p* < 0.05, compared with the Wistar Kyoto rat (WKY) group. ELE, *Eucommia* leaf extract.

NO is produced from arginine by endothelial NO synthase (eNOS) in the vascular endothelium and is responsible for ACh-induced relaxation. Therefore, we examined the plasma NO levels to evaluate the improvement in endothelial function following ELE treatment (Figure 3). The plasma NO level in the SHR-control group was significantly increased compared with that in the WKY group (6.37 ± 0.59 and 3.49 ± 0.22 μM, respectively; *p* < 0.05). The NO level in the SHR-ELE group (11.75 ± 1.95 μM) was significantly increased compared with that in the SHR-control group.

### 2.4. Measurement of Aortic Media Thickness

**Figure 4:**
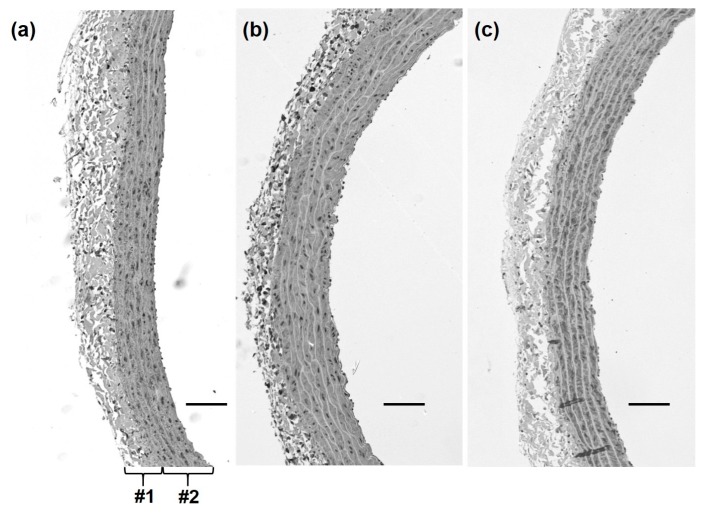
Representative histological alpha smooth muscle actin-stained images of the thoracic aorta of rats in the (**a**) Wistar Kyoto rat; (**b**) spontaneously hypertensive rat (SHR)-control; and (**c**) SHR-*Eucommia* leaf extract (ELE) groups. #1: arterial wall; #2: arterial media; scale bars = 100 μm.

**Figure 5:**
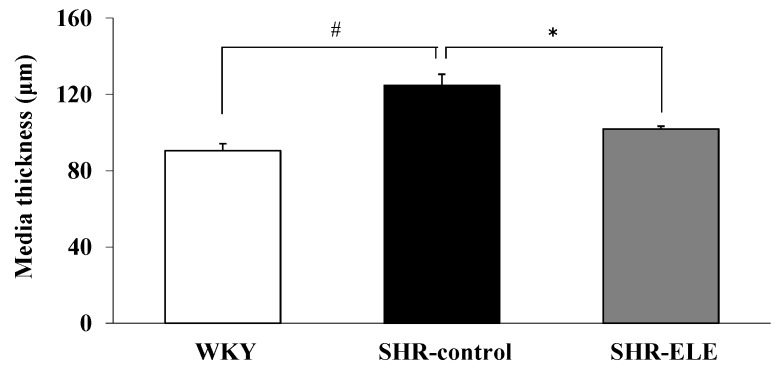
Thoracic aorta ring media thickness in the indicated treatment groups. The values represent the means ± SE; * *p* < 0.05, as compared with the spontaneously hypertensive rat (SHR)-control group; ^#^ *p* < 0.05, as compared with the Wistar Kyoto rat (WKY) group. ELE, *Eucommia* leaf extract.

We evaluated the long-term effects of ELE on endothelial function by determining the morphological changes in the blood vessels. Representative histology images of each group are shown in Figure 4a–c. In the WKY group, the arterial wall configuration appeared normal and the vascular smooth muscle cells (VSMCs) in the arterial media were arranged in an orderly manner. In contrast, the arterial wall in the SHR-control group appeared to have degenerated and showed hypertrophic VSMCs. The aortic tissue in the SHR-ELE group, however, seemed to be less morphologically altered than that of the SHR-control group. The media thickness in the SHR-control group was significantly greater than that in the WKY group (124.7 ± 5.9 and 90.4 ± 3.6 μm, respectively; *p* < 0.05). The arterial media thickness was significantly decreased in the SHR-ELE group (101.9 ± 1.4 μm, *p* < 0.05), as compared with that in the SHR-control group (Figure 5).

## 3. Discussion

In the present study, we investigated the restorative effect of chronic ELE treatment on vessel endothelial function, including the suppression of arterial media thickening, in SHRs. After seven weeks of treatment, ACh-induced aortic ring relaxation was significantly attenuated in the SHRs, as compared with the WKYs; however, no difference in SNP-induced vasorelaxation was observed among these three groups. These results indicated that vascular endothelial dysfunction had occurred in the SHR aortas. These results were consistent with those of a previous study showing that SHR exhibited reduced activity and expression of eNOS in the aorta [25] and that ACh-induced vasorelaxation was more impaired in SHRs than in WKYs [26]. Furthermore, the study indicated that the middle-aged SHR provided an appropriate animal model of vessel endothelial function and its therapeutic improvement. The sensitivity to ACh-induced vasorelaxation was significantly improved in the SHR-ELE group, as compared with the SHR-control group, suggesting that ELE treatment improved endothelium-dependent vasorelaxation by normalizing endothelial cell function. In addition, the plasma NO level in the SHR-ELE group was significantly higher than that in the SHR-control group, suggesting that ELE improved NO production by the vascular endothelial cells. NO is a major endothelium-dependent vasodilator of large blood vessels, including the thoracic aorta [27]. Therefore, these results indicated that this seven-week ELE treatment attenuated the endothelial dysfunction observed in middle-aged SHRs and thus improved the vascular function of the thoracic aorta.

In this study, the SHR-control group had higher basal plasma NO level than the WKY group. This finding was consistent with past reports showing that the higher plasma NO level in the SHR-control group could reflect an increased basal expression of inducible NO synthase (iNOS) [28], and oxidative stress-induced activation of iNOS [25]. SHRs exhibit increased NAD(P)H oxidase activity in vessel endothelial cells and the resultant oxidative stress induces iNOS [29], while reducing NO bioavailability, leading to endothelial dysfunction [30]. On the other hand, increased eNOS activity and reduced NADPH oxidase activity was associated with an improvement in vascular endothelial function in SHR [31], and increasing the expression and activation of eNOS elevated the plasma NO level in SHRs [32]. Therefore, the increased plasma NO level in the SHR-ELE group might be caused by a restoration of vascular endothelial cells by ELE treatment. The present study did not directly investigate the expression and activation of iNOS or eNOS and further studies are therefore necessary to investigate whether these were involved in the elevation of plasma NO level.

In addition, the inhibitory effect of ELE treatment on vascular hypertrophy might relate to the increased NO level. SNP-induced relaxation of the thoracic aorta did not differ significantly among the three study groups, which suggested that their mesothelial function did not differ during the study period. However, the media was significantly thicker in the SHR-control group than in the SHR-ELE group. In addition to its vasorelaxant effects, NO has a pivotal role in suppressing the abnormal proliferation of VSMCs [33]. Increased NADPH oxidase expression in the aortic VSMCs in SHRs impairs NO production and bioavailability [34], which most likely explains the thickened arterial media we observed in the SHR-control group. Indeed, ELE treatment may increase the aortic production and bioavailability of NO in the SHR-ELE group and suppress VSMC proliferation, thereby reducing media thickness. We assume that the NO level and bioavailability in the SHR-ELE group was high enough to prevent vascular hypertrophy, while the lower NO level and bioavailability in the SHR-control group was insufficient to afford such protection. Collectively, these results suggested that chronic treatment of SHRs with ELE restored vascular endothelial function, increasing the NO level and bioavailability, which attenuated vascular hypertrophy.

ELs contain compounds including the iridoid glucosides, geniposidic acid (GEA), and asperuloside (ASP); the caffeic acid derivative, chlorogenic acid (CHA) [35,36]. It has been reported that phytochemical components of ELs were 5.473% of GEA, 1.750% of ASP, 0.350% of CHA, and the flavonoids, 0.215% of quercetin and 0.023% of rutin [37]. The antihypertensive effect of ELE in SHRs has been attributed to GEA [21], which is one of the major active constituents in EL [35]. In addition, a single oral administration of GEA (140 mg/kg) significantly reduced blood pressure in SHR/Izm [38]. GEA has also been reported to enhance the oxidative defense system [39], although GEA and ASP have not been reported to show effective direct antioxidant activities. CHA has *in vitro* antioxidant activity [40] and has been reported to produce significant antihypertensive effects and to reduce oxidative stress in SHR at a dose of 300 mg/kg/day [41]. Quercetin and rutin also have *in vitro* antioxidant activity [40], however the levels of these compounds in ELs were estimated to be too small to account for the effects. These findings indicate that GEA might be the major active constituent responsible for the restoration of vascular function during chronic administration of ELE.

To our knowledge, there are no reports of toxic or negative health effects of ELE or ELE-containing foods. In this study, each individual rat in the SHR-ELE group ingested 60.5 mg of GEA per day for seven weeks and we did not observe any sign of toxicity.

In conclusion, to the best of our knowledge, this study is the first to demonstrate that long-term ingestion of ELE effectively improved the aortic endothelium- and NO-dependent relaxation, as well as increasing the plasma NO level, thereby decreasing vascular hypertrophy in SHRs. Therefore, ELE treatment may be effective in restoring vascular function.

## 4. Experimental Section

### 4.1. Reagents

Ethanol (EtOH), magnesium sulfate (MgSO_4_), monobasic potassium phosphate (KH_2_PO_4_), potassium chloride (KCl), sodium chloride (NaCl), sodium hydrogen carbonate (NaHCO_3_), SNP, and xylene were purchased from Kanto Chemical Co., Inc. (Tokyo, Japan). ACh, 10% buffered formalin, PE, papaverine (PV), ethylenediaminetetraacetic acid (EDTA) 2K, and 30% hydrogen peroxide were purchased from Wako Pure Chemical Industries (Osaka, Japan). Citraconic anhydride (0.05%, ImmunoSaver, Wako, Osaka, Japan), 10% normal donkey serum, hematoxylin, and diaminobenzidine were purchased from Nisshin EM (Tokyo, Japan) and Abcam (Cambridge, UK), Dojindo Laboratories (Kumamoto, Japan), and Merck KGaA (Darmstadt, Germany), respectively.

### 4.2. Animals

Five-week-old male SHR/Izm (*n* = 14) and WKY/Izm (*n* = 7) were purchased from Japan SLC, Inc. (Hamamatsu, Japan). They were housed in the Animal Research Center of the Faculty of Agriculture at Shinshu University under a controlled ambient temperature of 23 ± 1.0 °C, with 50% ± 5% relative humidity and a 12-h light/dark cycle. After an adaptation period of one week, six-week-old rats were used for the experiments. All animal experiments were carried out in strict accordance with the recommendations of the standards relating to the Care and Management of Laboratory Animals and Relief of Pain (2006) published by the Japanese Ministry of the Environment. The protocol was approved by the Animal Care Committee of the Faculty of Agriculture at Shinshu University (permit number: 250043). The surgeries were performed under diethyl ether anesthesia, and all efforts were made to minimize animal suffering and discomfort.

### 4.3. Preparation of ELE

The ELs were collected from the Sichuan District of China. ELE was prepared using a previously described method [42]. Briefly, fresh ELs were treated with steam at 100–110 °C, dried, and roasted. Two tons of the roasted leaves were steeped in 10 tons of hot water at 90 °C for 1 h, and the resulting extract was filtered and concentrated. The concentrate was left to stand for 1 day, after which it was filtered, further concentrated, vacuum-dried, and powdered to obtain a yield of 18%. We measured each of the major components (GEA, ASP, and CHA) in this ELE by high-performance liquid chromatography [18]. ELE comprised GEA 65.8 mg/g, ASP 11.4 mg/g, and CHA 39.7 mg/g.

### 4.4. Long-Term Administration of ELE

A moderate-fat (MF) diet (Oriental Yeast Company Ltd., Tokyo, Japan) was used as the control diet. The MF diet consisted of water (7.8%), proteins (23.1%), lipids (5.1%), ash (5.8%), dietary fiber (2.8%), and nitrogen-free extract (55.3%). The test diet was prepared by adding 5% (*w*/*w*) ELE to the MF diet. One day before the experiment, the SHRs were divided into two groups (*n* = 7/group, six-week-old) that were fed either the MF diet (SHR-control group) or the MF diet with 5% (*w*/*w*) ELE (SHR-ELE group). The WKY rats (*n* = 7, six-week-old) were fed the MF diet (WKY group). The effects of ELE were determined during a seven-week *ad libitum* administration. Rat body weights and their food and water intakes were measured twice a week throughout the experimental period. SBP was measured using the tail cuff method with a Softron BP98A (Softron Co. Ltd., Tokyo, Japan) at one day before (day-1), and three and seven weeks after, the beginning of the experiment.

### 4.5. Measurement of Endothelial Function Using Thoracic Aorta Rings

The endothelial function of the thoracic aorta of each rat was measured at the end of the experimental period. The assay was performed according to the method of Nakamura *et al.* [43]. Briefly, each rat (*n* = 7/group) was anesthetized with diethyl ether and subsequently exsanguinated via the abdominal aorta. The isolated thoracic aorta was cut into 2-mm rings and placed in cold Krebs–Henseleit buffer (119 mM NaCl, 4.7 mM KCI, 1.1 mM KH_2_PO_4_, 1.2 mM MgSO_4_, and 25 mM NaHCO_3_, pH 7.4). The endothelium was removed from some of the rings by gently rubbing the intimal surface with a small pair of forceps, to investigate the involvement of endothelium-derived NO in the subsequent measurements. The rings were mounted at a resting tension of 1.5 g in a 5 mL organ bath (UFER UC-05A Micro-easy Magnus system, Kishimoto Medical Instruments, Kyoto, Japan) containing warmed (37 °C) and oxygenated (O_2_:CO_2_, 19:1) Krebs-Henseleit buffer. After a 60 min equilibration at this tension, vasoconstrictor PE (0.3 μM) was added to the rings. When a constriction plateau was reached, the removal of the endothelium was confirmed by applying 100 μM ACh to the constricted rings. After checking the endothelial condition, the tissues were constricted again with PE. Upon attaining a constriction plateau, endothelium-dependent and -independent relaxation was evaluated using the concentration-response curves for ACh (10^−9^–10^−4^ M) in the endothelium-intact rings and those of SNP (10^−11^–10^−5^ M) in the endothelium-denuded rings. The vasorelaxant effect was expressed as the percentage relaxation of PE-induced constriction. At the end of the evaluation of endothelial function using ACh, the rings were completely dilated by the addition of excess vasorelaxant (100 μM PV) to confirm that the mesothelium functioned properly.

### 4.6. Quantification of NO in Rat Plasma

The blood samples collected during exsanguination were transferred to a tube containing 10% EDTA-2K anticoagulant. The uncoagulated blood was centrifuged at 1811× *g* for 10 min at 4 °C (Eppendorf 5810R; Eppendorf AG, Hamburg, Germany) to separate the plasma. The total NO (nitrite and nitrate) in the plasma was determined using the Griess reaction. A nitrate/nitrite colorimetric assay kit, where nitrate was converted to nitrite by nitrate reductase, was employed according to the manufacturer’s instructions (Cayman Chemical Company, Ann Arbor, MI, USA). The measurements were performed in triplicate and the nitrate/nitrite concentrations (μM) were expressed as the means ± standard error (SE).

### 4.7. Immunohistochemical Analysis to Visualize Thoracic Aorta Smooth Muscle Cells

To visualize the smooth muscle cells of the aorta, the sections were stained using an avidin-biotin-peroxidase complex method. Briefly, the thoracic aorta of each rat was isolated as described in Section 4.5. The aorta was immediately fixed in 10% buffered formalin. Paraffin-embedding of the fixed tissues was performed at the Research Center for Human and Environmental Science at Shinshu University. The 4-μm thick paraffin-embedded tissue sections were deparaffinized with xylene, rehydrated with EtOH, and antigen retrieval was performed in the ImmunoSaver solution for 50 min at 98 °C. The sections were treated with 3% hydrogen peroxide solution for 30 min, blocked for 30 min with 10% normal donkey serum, and were subsequently incubated overnight at 4 °C with polyclonal rabbit anti-alpha smooth muscle actin antibody (1:1000; Abcam). The sections were subsequently treated with horseradish peroxidase-conjugated secondary antibody (1:500; Abcam) for 30 min at room temperature and were stained with diaminobenzidine for 1 min. After counterstaining with hematoxylin, the tissues were thoroughly washed with running tap water. The stained sections were dehydrated with EtOH and xylene and coverslipped on a microscopy slide. The thickness of the intra media—consisting of smooth muscle cells—was measured on the prepared slides under a microscope (Axio Imager A1; Carl Zeiss AG, Oberkochen, Germany) using AxioVision software (Carl Zeiss AG).

### 4.8. Statistical Analysis

All data were expressed as the means ± SE. The statistical significance of differences between two groups was estimated using Student’s *t*-test for unpaired observations. *p* < 0.05 was considered statistically significant.

## 5. Conclusions

The results of this study demonstrate that chronic administration of ELE effectively restored the aortic endothelium-dependent, NO-mediated relaxation, and the plasma NO level, which decreased arterial media thickening in SHRs. These data suggest that ELE may exert anti-endothelial dysfunction and anti-vascular hypertrophy effects.
